# Using an untargeted metabolomics approach to analyze serum metabolites in COVID-19 patients with nucleic acid turning negative

**DOI:** 10.3389/fphar.2022.964037

**Published:** 2022-08-24

**Authors:** Wenyu Chen, Ming Yao, Miaomiao Chen, Zhao Ou, Qi Yang, Yanbin He, Ning Zhang, Min Deng, Yuqi Wu, Rongchang Chen, Xiaoli Tan, Ziqing Kong

**Affiliations:** ^1^ Department of Respiration, Affiliated Hospital of Jiaxing University, Jiaxing, China; ^2^ Department of Anesthesiology and Pain Research Center, Affiliated Hospital of Jiaxing University, Jiaxing, China; ^3^ Key Laboratory of Digital Technology in Medical Diagnostics of Zhejiang Province, Hangzhou, China; ^4^ Department of Hepatic Surgery, Fudan University Shanghai Cancer Center, Shanghai, China; ^5^ Department of Infection, Affiliated Hospital of Jiaxing University, Jiaxing, China; ^6^ Calibra Lab at DIAN Diagnostics, Hangzhou, China

**Keywords:** COVID-19, SARS-CoV-2, metabolomic, metabolites, mass spectrometry, serum

## Abstract

**Background:** The coronavirus disease of 2019 (COVID-19) is a severe public health issue that has infected millions of people. The effective prevention and control of COVID-19 has resulted in a considerable increase in the number of cured cases. However, little research has been done on a complete metabonomic examination of metabolic alterations in COVID-19 patients following treatment. The current project pursues rigorously to characterize the variation of serum metabolites between healthy controls and COVID-19 patients with nucleic acid turning negative *via* untargeted metabolomics.

**Methods:** The metabolic difference between 20 COVID-19 patients (CT ≥ 35) and 20 healthy controls were investigated utilizing untargeted metabolomics analysis employing High-resolution UHPLC-MS/MS. COVID-19 patients’ fundamental clinical indicators, as well as health controls, were also collected.

**Results:** Out of the 714 metabolites identified, 203 still significantly differed between COVID-19 patients and healthy controls, including multiple amino acids, fatty acids, and glycerophospholipids. The clinical indexes including monocytes, lymphocytes, albumin concentration, total bilirubin and direct bilirubin have also differed between our two groups of participators.

**Conclusion:** Our results clearly showed that in COVID-19 patients with nucleic acid turning negative, their metabolism was still dysregulated in amino acid metabolism and lipid metabolism, which could be the mechanism of long-COVID and calls for specific post-treatment care to help COVID-19 patients recover.

## Introduction

The acute respiratory syndrome coronavirus 2 (SARS-CoV-2) infection has caused a major threat to worldwide public health, and the new kind of coronavirus pneumonia (COVID-19), an acute respiratory infectious disease, is caused by this infection (Rahimkhoei et al., 2021). According to the World Health Organization, over 435 million cases and 5.9 million fatalities have been reported worldwide as of 1 March 2022 (WHO 2022).

Fever or chills, chronic dry cough, shortness of breath or difficulty in breathing, muscle or body aches, headache, new loss of taste or smell, and gastrointestinal problems are all signs of COVID-19 (To et al., 2021). COVID-19 is classed as mild or moderate in 81 percent of those infected, and 19 percent of those diagnosed will develop multiple organ failure (Wu and McGoogan, 2020). SARS-CoV-2 is a systemic disease that affects numerous organs, including kidneys, muscles, lymph nodes, gastrointestinal organs, and others (Soni et al., 2021), and causes many long-term health problems (Crook et al., 2021).

Currently, many multi-omics studies on COVID-19 patients have been conducted, revealing the underlying mechanism of the virology, pathogenesis, host response, etc. of COVID-19 infection at molecular and cellular levels (Shen et al., 2020; Su et al., 2020; Valdes et al., 2022). These studies have deepened our understanding of the fundamentals of COVID-19 infection and aided in the prevention, control, and treatment of COVID-19. Nevertheless, most current studies have focused on COVID-19 patients who were unconcerned about disease severity and were most likely in the early stages of infection. As a result, little is known about the metabolic status of COVID-19 patients in the late stages of infection. A recent proteomic and metabolomic study revealed that COIVD-19 caused persistent abnormalities after discharge (Li et al., 2022), emphasizing the importance of a study focused on cured COIVD-19 patients.

In this study, we used untargeted metabolomics to analyze the metabolomic differences between COVID-19 patients with nucleic acid turning negative (whom are abbreviated as cured COVID-19 patients in subsequent paragraphs of the article) and health controls. We aim to reveal the metabolic status of COVID-19 patients so that we can better monitor the health of COVID-19 survivors in the future to alleviate the possible post-COVID sequela.

## Methods and analysis

### Study cohort

This study included a total of 20 COVID-19 patients who were hospitalized in Jiaxing Hospital from January to March 2020 The inclusion criteria for cured COVID-19 patients are: 1) the disappearance of major clinical signs and 2) have two consecutive negative result from COVID-19 nucleic acid tests (CT ≥ 35). All patients were sampled after fulfilling these 2 criteria but not yet discharged from hospital. The Diagnosis and Treatment Protocol for COVID-19 Patients 8th edition was used for the diagnose and treatment of COVID-19 patients (Tentative 8th Edition). Nucleic acid was extracted from sputum or a throat swab using automatic nucleic acid extraction and purification device (GENFINE, Jiangsu Changzhou, China, P961002) and viral nucleic acid extraction reagent (GENFINE, Jiangsu Changzhou, China, Y502-G40). Nucleic acid identification was made using fluorescence quantitative PCR (Thermo Fisher Scientific, ABI7500) and a SARS-CoV-2 nucleic acid detection kit (Wuhan Easy Diagnosis Biomedicine Co., Ltd., China, 2019-nCaV-100-02). Twenty healthy individuals were also included as a control.

Serum collecting tubes (Chengdu Puth Medical Plastics Packaging Co., Ltd., China) were used to obtain the blood samples. Furthermore, the sample was centrifuged for 10 min at 1,500 g. The serum samples were then transferred to new storage tubes and stored at −80 °C immediately.

The samples in this investigation came from a clinical trial that our team started and registered as MR-33-20-004032 in the Medical Research Registration and Filing Information System. Jiaxing Hospital’s study has been approved by the Ethical/Institutional Review Board. All patients before participation gave informed written consent.

### Sample preparation for metabolome analysis

The metabolomics analysis and sample preparation were carried out as previously described (Shen et al., 2020). To inactivate potential viruses, ethanol was added to the serum samples and shaken before drying in a biosafety hood. After being dried, the samples were then subjected to downstream processing in preparation for metabolomics analysis. In brief, a 300 μl methanol extraction solution was added to 100 μl deactivated serum samples and shaken for 2 min. Centrifugation at 4,000 × g for 10 min was then performed to denature and precipitate proteins. To ensure comprehensive and accurate metabolomic analysis, each supernatant was divided into four fractions and analyzed using a nontarget metabolomic platform consists of 4 different metabolomic analytical methods: two for analysis by using two separate reverse-phase/ultra-performance liquid chromatography (RP/UPLC)-MS/MS methods with positive ion-mode electrospray ionization (ESI); one for analysis by using RP/UPLC-MS/MS with negative-ion mode ESI; one for analysis using hydrophilic interaction liquid chromatography (HILIC)/UPLC-MS/MS with negative-ion mode ESI. To remove the organic solvent, four fractions were all dried with nitrogen gas, and then re-dissolved in four different reconstitution solvents which were compatible with the 4 UPLC-MS/MS methods respectively.

### Untargeted UPLC–MS/MS analysis

All UPLC-MS/MS methods were conducted using the ACQUITY 2D UPLC system (Waters, Milford, MA, United States) and Q Exactive HF hybrid Quadrupole-Orbitrap system (Thermo Fisher Scientific, San Jose, United States) with HESI-II heated ESI source and Orbitrap mass analyzer. The mass spectrometer was operated at a resolution of 35,000 mass units (200 m/z). In the first UPLC-MS/MS method, the QE was operated under positive electrospray ionization (ESI) and a C18 column (UPLC BEH C18, 2.1 × 100 mm, 1.7 μm; Waters) was used in UPLC. Water and methanol containing 0.05% perfluorooctanoic acid (PFPA) and 0.1% formic acid (FA), with a final pH at 3 were used as mobile solutions for gradient elution. When the polar mobile phases increased from 5 percent to 95 percent, the gradient elution for techniques with C18 columns was performed in a 7-min run. The QE was still under ESI positive mode for the second method, and the UPLC employed the same C18 column as in the first. The mobile phase solutions were composed of water, acetonitrile, methanol, 0.01% FA, and 0.05% PFPA at pH 3, optimized for more hydrophobic compounds. As to the third UPLC-MS/MS method, the QE was performed under negative ESI mode. A C18 Column was used and it was eluted with mobile solutions that contained methanol and water in 6.5 mM ammonium bicarbonate at pH 8. In terms of the fourth UPLC-MS/MS method, the HILIC UPLC column (UPLC BEH Amide, 2.1 × 150 mm, 1.7 μm; Waters) was used, and the mobile solutions were composed of water and acetonitrile with 10 mM ammonium formate at pH 10.8, and the gradient elution is conducted in 7 minutes run with the polar mobile phase decreased from 80% to 20%. The QE was performed under negative ESI mode. The QE mass spectrometer analysis was carried out by alternating MS and data-dependent MS2 scans using dynamic exclusion. The scan range was 70–1,000 m/z, and the MS capillary temperature was 350°C, sheath gas flow rate was at 40, and aux gas flow rate was at 5 for both positive and negative methods.

### Quality control of metabolome analysis

A mixture of internal standards was spiked into every sample to aid chromatographic peak alignment and instrument stability monitoring. Instrument variability was determined by calculating the median relative SD (RSD) of all internal standards in each sample. The median RSD of this study is ≤5%, which fulfilled our QC criteria. In addition, extracted water samples served as blanks, and extracted commercial plasma samples were used for monitoring instrument variation.

### Compound identification

The identification of metabolites followed the pre-processing of raw data, peak finding/alignment, and peak annotation using in-house software. Metabolites were identified by searching the in-house database which included over 3,300 standards whose entries were generated by running purified compound standards on the experimental platforms. The identification must meet three criteria well (Shen et al., 2020; Bi et al., 2022): 1) narrow window retention time (variation less than 0.1 min), 2) accurate mass with variation less than 10 ppm, and 3) MS/MS spectra with high forward and reverse scores that stemmed from the comparison of the ions present in the experimental spectrum to those present in the library spectrum entries. By following these three criteria, almost all isomers can be distinguished. All detected metabolites meet the level 1 requirements by the Chemical Analysis Working Group (CAWG) of the Metabolomics Standards Initiative (MSI) (Sumner et al., 2007), except a few asterisk labeled lipids that were matched to in silico MS/MS spectral.

### Statistical analysis

R software (version 3.6.1) was used for data analysis (Lu et al., 2021). The Kolmogorov-Smirnov test was used to determine whether the data distributions were normal. The mean (±standard deviation) was used to represent normally distributed data, whereas the median (±interquartile range) was used to represent abnormally distributed data, and categorical variables were represented as frequencies (%). Student’s t-test or Mann-Whitney test (for continuous data) and Fisher’s exact test or chi-squared test (for categorical data) were used to examine differences across groups. The unpaired two-sided student t-test was performed to determine statistical significance, and the adjusted *p*-value was obtained using Benjamini & Hochberg adjustment (Adjusted *p*-value < 0.05).

The critical variables with discriminative power were identified using a supervised method called partial-least squares discrimination analysis (PLS-DA). The multiple correlation coefficient (R2) was used to validate PLS-DA models. Following that, we used cross-validation to obtain the cross-validated R2 (Q2), as well as permutation tests using 2000 iterations (*p* < 0.001). By using variable importance in projection (VIP), the relative relevance of each metabolite to the PLS-DA model was determined. We can use the *p*-value or the fold change of univariate analysis with the variable import in point (VIP) of the PLS-DA model to screen for distinct metabolites further.

To screen for differential metabolites, the VIP value was paired with the *p*-value or fold change of univariate analysis. The following are the screening criteria: 1) Metabolites with fold change ≥1.5 or fold change ≤0.67; 2) Metabolites with a *p*-value (adjusted by BH) < 0.05; 3) Metabolites with VIP ≥1. The metabolite differed considerably between the groups if the above three requirements were met. The pheatmap R packages were used to build the heatmap. We used the Euclidean distance measure and the ward clustering algorithm to make the heatmap. What’s more, we compared the groups based on the differentially altered metabolites, and we investigated the metabolomic pathways influenced by the COVID-19 infection using a KEGG pathway analysis. R cluster Profiler (v3.12.0) package with BH multiple comparison test as FDR <0.1 was used to determine significant enriched KEGG pathways that were enriched for at least three metabolites (for significantly altered metabolites), FDR <0.1, and fold enrichment >2.

## Results

### Characteristics of patients

The First Hospital of Jiaxing took serum blood samples from 20 COVID-19 patients and 20 healthy volunteers. The COVID-19 patients were 47.9 ± 13.6 years old on average, with 6 (30.0%) of them being female. The control group’s average age was 46.7 ± 12.9 years, with 6 (30.0%) of the participants being female. Platelet count, white blood cell count, monocyte count, lymphocyte count, alanine aminotransferase (ALT), glutamyl transferase (GGT), aspartate aminotransferase (AST), direct bilirubin (DBIL), total bilirubin (TBIL), creatinine, albumin (ALB), and lactic acid were among the 12 clinical indicators measured. Compared with healthy controls, COVID-19 patients had higher monocyte counts (*p* = 0.00014), lower total lymphocyte counts (*p* = 0.0017), lower albumin concentrations (*p* = 0.00015), lower TBIL (*p* = 0.00014), lower DBIL (*p* = 0.0089), and the rest showed no significant difference.

### Composition of serum metabolites

**FIGURE 1:**
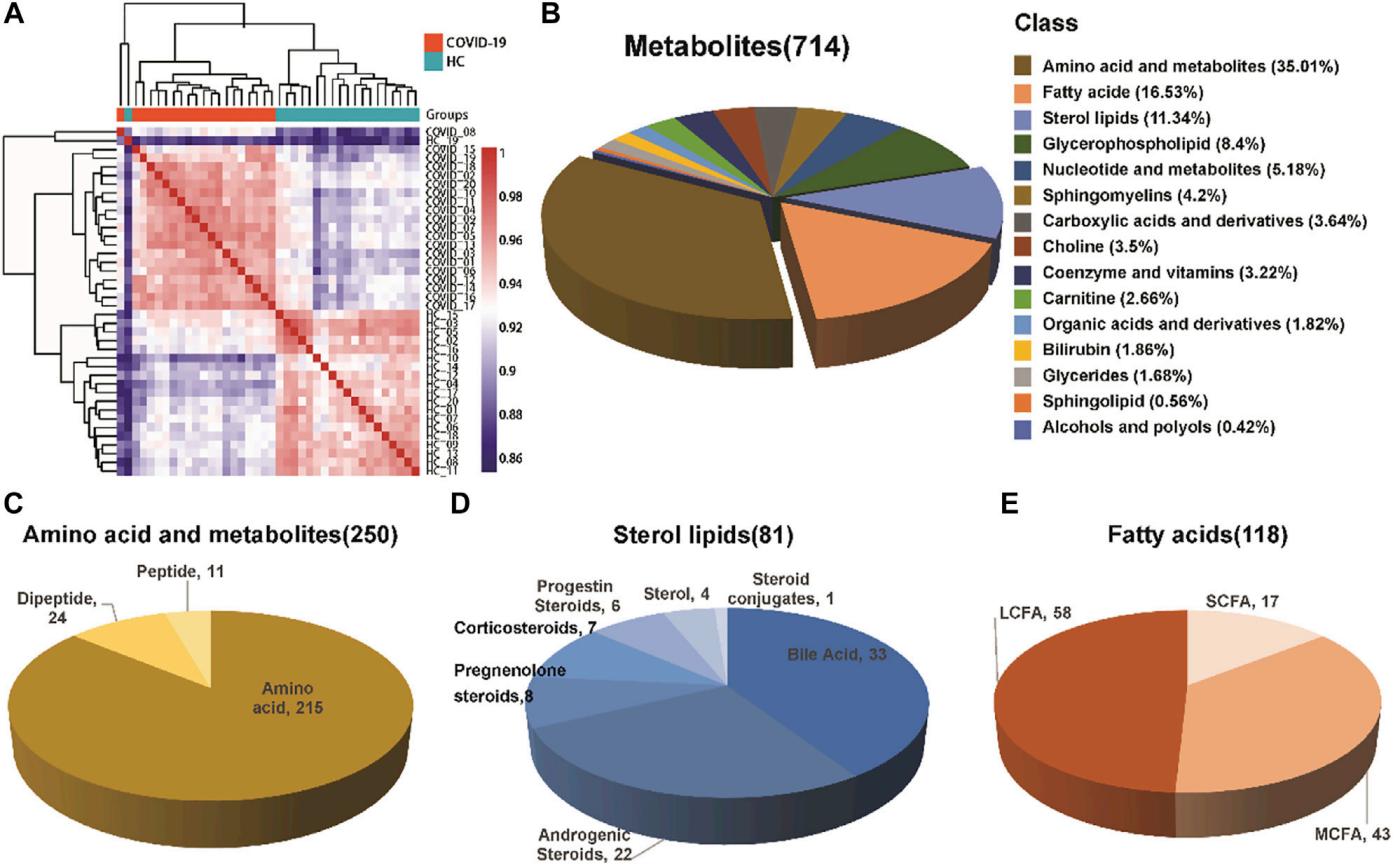
Composition of serum metabolites. **(A)** Correlation coefficient test between the healthy control group and COVID-19 group. **(B)** Classification and proportion of metabolites. **(C–E)** Variety of major metabolites, including amino acids and their metabolites, fatty acids, and sterol lipids.

A total of 714 metabolites were detected from 40 serum samples using untargeted metabolomics analysis, as shown in Table 2. A Spearman correlation coefficient test (de Winter et al., 2016) was used to analyze the metabolite-metabolite correlation among identified metabolites in healthy controls and COVID-19 patients. A heatmap was used to show the correlations between groups in the form of a matrix in Figure 1A. We noticed that the COVID-19 group is distinct from the healthy control group.

**TABLE 1:** Baseline characteristics of COVID-19 patients and healthy controls.

Participant characteristics	Healthy controls (*n* = 20)	COVID-19 patients (*n* = 20)	*p-value*
Demographics			
Age (years)	46.7 ± 12.9	47.9 ± 13.6	0.786
Gender			
Male	14	14	1
Female	6	6	
BMI	23.7 ± 2.8	23.2 ± 3.2	0.391
Clinical characteristics			
White blood cell (×10^^9^/L)	5.66 ± 1.75	5.93 ± 1.77	0.9
Lymphocytes (×10^^9/^L)	1.93 ± 0.59	1.35 ± 0.32	0.00032
Monocytes (×10^^9^/L)	0.28 ± 0.08	0.83 ± 1.60	0.00014
Platelets (×10^^9^/L)	229.05 ± 46.09	244.67 ± 61.62	0.36
Alanine aminotransferase (IU/L)	35.8 ± 38.39	64.85 ± 91.56	0.14
Aspartate aminotransferase (IU/L)	26.75 ± 17.76	33.95 ± 32.510	0.053
Creatinine (μmol/L)	79.86 ± 13.19	76.7 ± 18.87	0.32
Glutamyltransferase (IU/L)	35.8 ± 32.02	41.88 ± 34.57	0.12
Total bilirubin (μmol/L)	15.27 ± 5.87	8.83 ± 1.89	0.0032
Direct bilirubin (μmol/L)	5.01 ± 4.03	2.26 ± 0.60	0.00086
Lactate dehydrogenase (IU/L)	222.5 ± 64.35	208.82 ± 32.64	0.71
Albumin (g/L)	50.28 ± 6.45	41.84 ± 5.58	0.00031

The *p* value of significantly differed clinical indexes are displayed in bold. Note: Data are shown as BMI, body mass index; mean ± SD. N, number of participants.

**TABLE 2:** Classification of metabolites.

Class	Numbers	Percentage (%)	
Amino acid and metabolites	Amino acid	215	30.11
	Dipeptide	24	3.36
	peptide	11	1.54
Fatty acids	SCFA	17	0.38
	MCFA	43	6.02
	LCFA	58	8.12
Sterol lipids	Bile acid	33	4.62
	Androgenic steroids	22	3.08
	Corticosteroids	7	0.98
	Pregnenolone steroids	8	1.12
	Progestin steroids	6	0.84
	Sterol	4	0.56
	Steroid conjugates	1	0.14
Glycerophospholipid	60	8.40	
Nucleotide and metabolites	37	5.18	
Sphingomyelins	30	4.20	
Carboxylic acids and derivatives	26	3.64	
Choline	25	3.50	
Coenzyme and vitamins	23	3.22	
Carnitine	19	2.66	
Organic acids and derivatives	13	1.82	
Bilirubin	13	1.82	
Glycerides	12	1.68	
Sphingolipid	4	0.56	
Alcohols and polyols	3	0.42	

MCFA, medium-chain fatty acids; SCFA, short-chain fatty acids; LCFA, long-chain fatty acids.

The identified metabolites were grouped into different chemical groups according to their metabolite classification, including amino acid and metabolite, fatty acid, sterol lipids, glycerophospholipid, nucleotide and metabolites (Figure 1B). We noticed that most of the identified metabolites belong to amino acid and metabolite (35.01%), fatty acid (16.53%) and sterol lipids (11.34%). These three chemical groups could be further divided into several different subgroups (Figures 1C–E), e.g., Sterol lipids mainly consist of bile acid and androgenic steroids, while fatty acids consist of short chain fatty acid, long chain fatty acid, and medium chain fatty acid.

### Untargeted metabolomics analysis of serum from subjects

**FIGURE 2:**
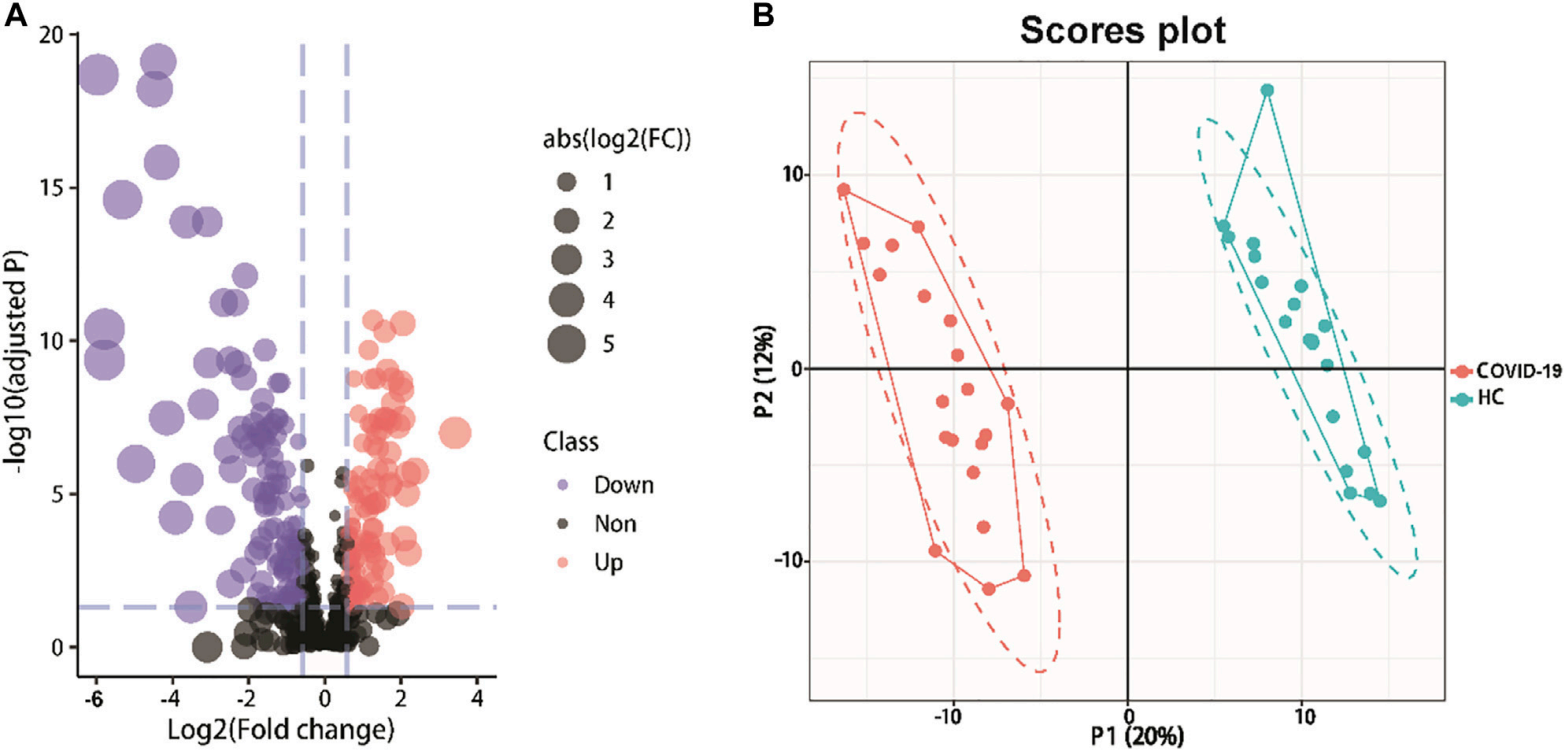
Detected metabolites analysis of serum samples from COVID-19 patients and healthy controls. **(A)**Volcano plots show the change in transformed P-adjust (−log10) against the log2 (COVID-19/Healthy). Blue dashed lines: cut-off values (ratio >1.5 and *p*-value adjust<0.05). Red dots: Highly expressed metabolites in COVID-19 patients (115). Purple dots: Low expression of metabolites (128). The size of the points varies according to the absolute value of [Log2(COVID-19/Healthy)]. **(B)** PLS-DA plot of differentially abundance metabolites. Red and blue points are COVID-19 patients and healthy controls, respectively (R2 = 0.994, Q2 = 0.921).

We created a supervised PLS-DA model that focused on the actual class discriminating variations to find metabolites that are distinctive between the two groups. The first three components’ goodness of fit (R2) and model prediction ability (Q2) for COVID-19 patients and healthy controls, respectively, were 0.994 and 0.921 (Figures 2A,B). The analysis revealed an appreciable difference between the metabolomic profile of COVID-19 patients and healthy controls.

### Metabolomic changes in COVID-19 patients serum patient characteristics

**FIGURE 3:**
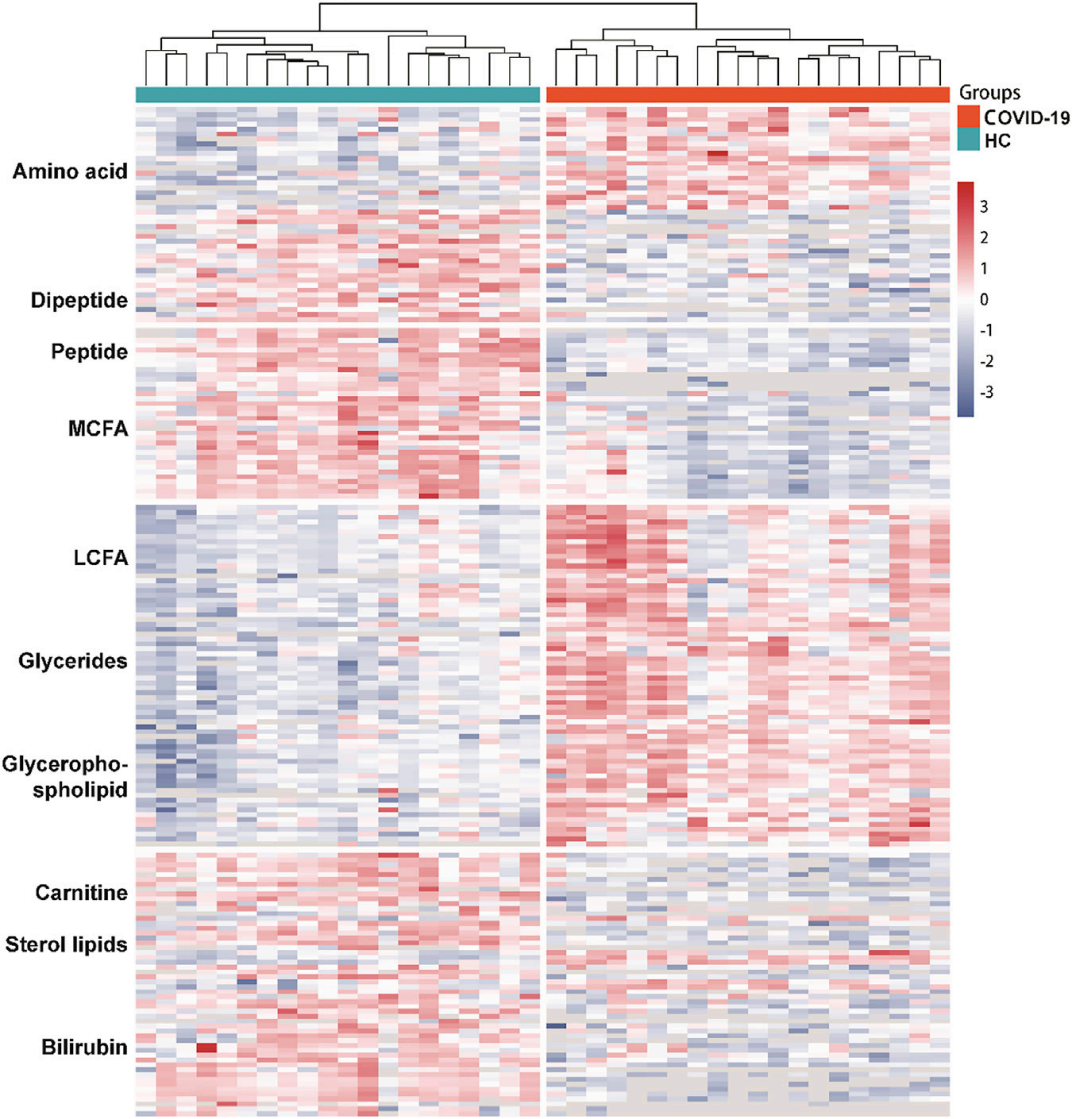
Dysregulated Metabolites in the serum of COVID-19 patients. Heatmap visualization of significantly altered metabolites between COVID-19 group and healthy controls. Metabolites included in the heatmap meet the requirement that fold-change >1.5 or <0.67 and adjust *p* value of <0.05. The color bar represents the relative intensity of identified proteins from −3 to 3.

The differential metabolites between groups were screened using multivariate statistical and univariate analysis. The alteration of serum metabolites was determined by VIP value combined with Student’s t-test and a fold change (FC), with VIP >1.0, *p* < 0.05, and FC ≥ 1.5 or ≤0.67 as the criterion of statistical significance. The above threshold led to 203 differential metabolites between COVID-19 Patients and healthy controls (Figure 3; Table 3). In COVID-19 patients, 21 amino acid metabolites were upregulated, while 37 were downregulated, particularly the hydroxyethyl amino acid derivatives (Supplementary Figure S1). In addition, the peptide metabolites in patients with COVID-19 were significantly reduced. Among the fatty acid-related metabolites, 6 long-chain fatty acids and 14 medium-chain fatty acids were down-regulated, while 22 long-chain fatty acids were up-regulated. Meanwhile, in COVID-19 patients, 18 glycerophospholipids were up-regulated. The majority of sterol metabolites were significantly down-regulated.

**TABLE 3:** Summary of different types of metabolites in COVID-19 patients.

Class	Up	Down	
Amino acid and metabolites	Amino acids	19	22
	Dipeptide	2	10
	peptide	\	5
Fatty acids	SCFA	1	1
	MCFA	\	14
	LCFA	22	6
Glycerides	12	\	
Glycerophospholipid		18	
Carnitine	1	8	
Sterol lipids	Bile Acid	2	4
	Androgenic Steroids	\	2
	Corticosteroids	\	5
	Progestin Steroids	\	2
	Sterol	\	2
Bilirubin	\	11	
Coenzyme and vitamins	4	5	
Carboxylic acids and derivatives	7	\	
Nucleotide and metabolites	1	6	
Choline	4	\	
Organic acids and derivatives	3	1	
Sphingomyelins	\	2	
Alcohols and polyols	1	\	

### Bioinformatics enrichment of dysregulated pathways

**FIGURE 4:**
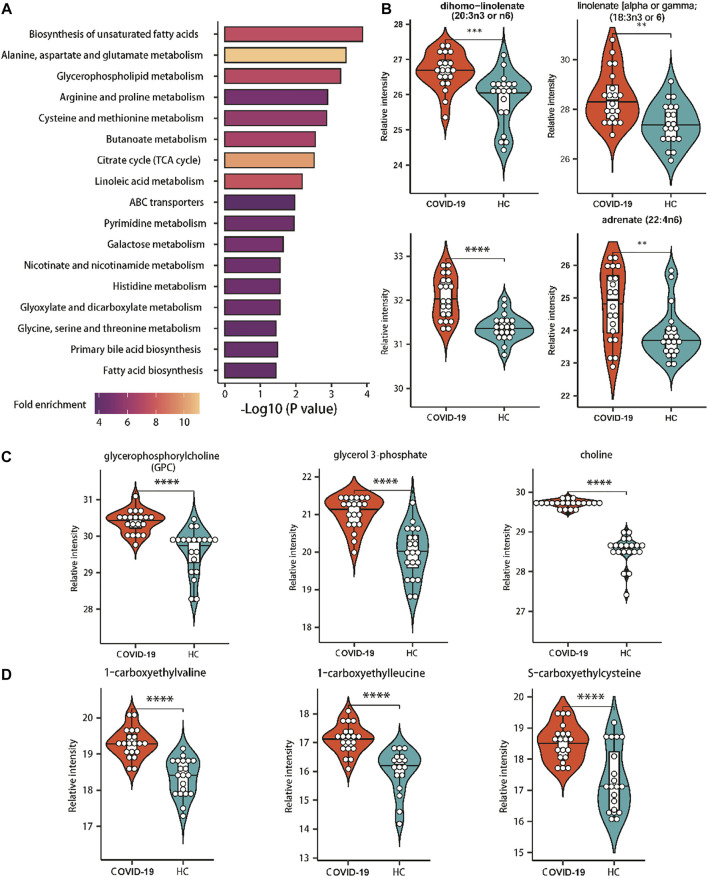
Enriched pathways of significantly altered metabolites of COVID-19 patients. **(A)** Metabolic pathways enriched based on metabolites consistently increased or decreased in COVID patients compared with healthy controls. Fold enrichment is represented by color intensity. One-sided Fisher’s exact test followed by BH multiple comparison test. **(B)** Relative abundance of metabolites involved in the biosynthesis of unsaturated fatty acids. **(C)** Relative abundance of metabolites involved in the glycerophospholipid metabolism. **(D)** Relative abundance of metabolites involved in the amino acids metabolism. ∗, *p*-value <0.05 ∗∗, *p*-value <0.01; ∗∗∗, *p*-value <0.001; ∗∗∗∗, *p*-value <0.0001.

According to the significantly changed metabolites, metabolite set enrichment analysis (MSEA) and pathway analysis were used to determine the altered metabolic pathways in COVID-19 patients. Pathway analysis revealed that 17 metabolic pathways were changed significantly in patients (Figure 4A). Unsaturated fatty acid biosynthesis, glycerophospholipid metabolism, and amino acid metabolism were covered (Figures 4B–D).

### Correlation between metabolic changes and clinical parameters

**FIGURE 5:**
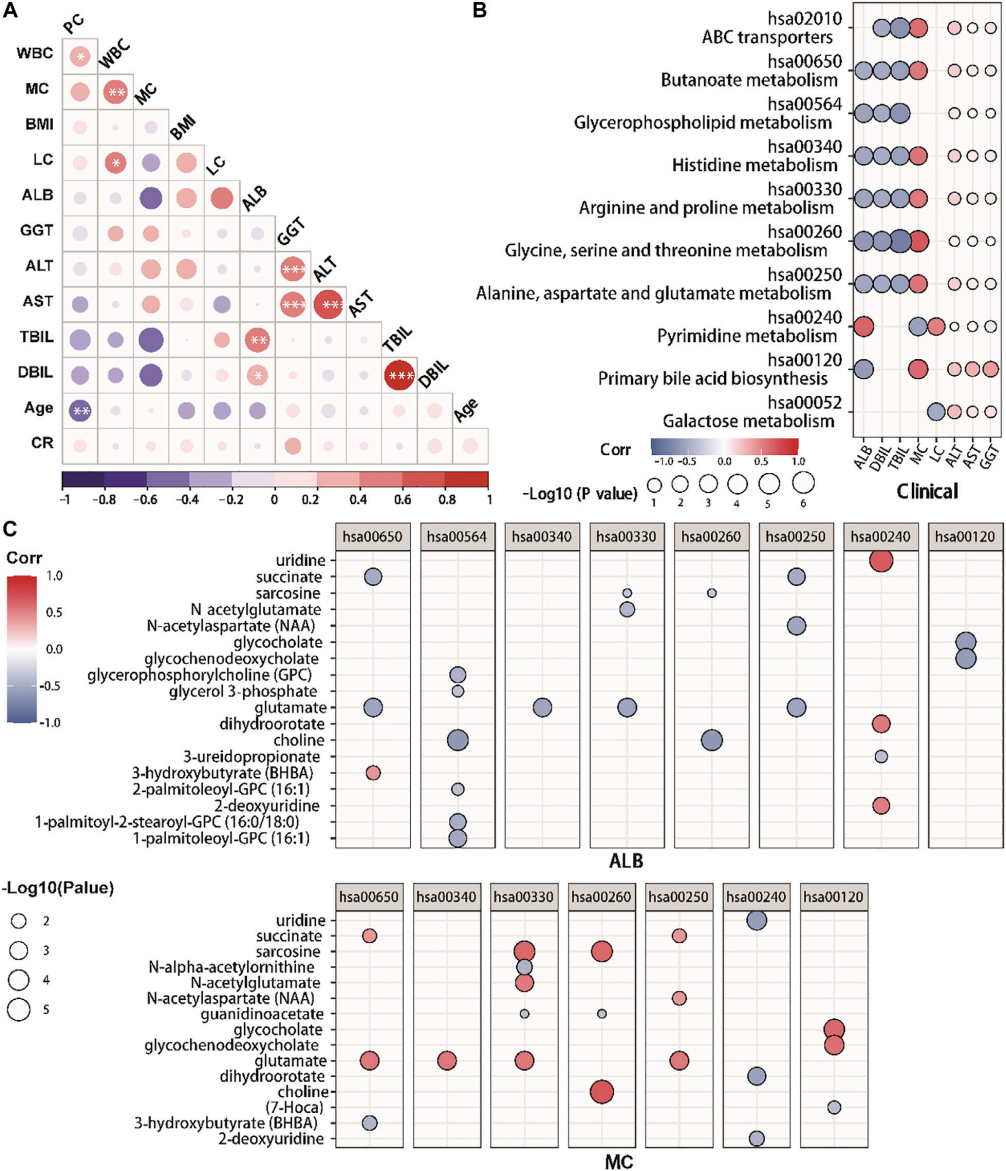
The correlation between clinical indexes and metabolites in COVID-19 patients. **(A)** Correlation matrix of 12 clinical features from hospitalized patients. The circle size corresponds to the absolute value of the Spearman rank correlation coefficient, with the red (blue) color indicating a positive (negative) correlation. ∗FDR <0.05, ∗∗FDR <0.01, ∗∗∗FDR <0.001. **(B,C)**. The altered metabolic pathways and clinical indexes correlations. **(C)** The altered metabolites and clinical indexes correlations. The circle size corresponds to the −log10 (*p*-value) of the Spearman rank correlation. Only correlations with |R|>0.5 and *p* < 0.05 were highlighted. With red (blue) color shows a positive (negative) correlation. PLC, peripheral lymphocyte count.

In this retrospective investigation, Spearman correlation analysis was conducted to find the correlation between clinical indices (Figure 5A). The results revealed that ALB was positively correlated with PYMPH, TBIL, and DBIL, and negatively correlated with monocytes, according to our findings (MC). Meanwhile, DBIL was positively correlated with TBIL. Furthermore, we examined correlations between various clinical indexes and metabolite pathways through Spearman correlation analysis. (Figure 5B). In COVID-19 patients, ALB was associated with amino acid metabolisms including serine, glycine, arginine, threonine metabolism, and proline histidine metabolism. Moreover, ALB was correlated with primary bilirubin acid biosynthesis and pyrimidine metabolism, consistent with the negative correlation between ALB and MC. The correlation between monocyte and various metabolic pathways is the opposite of ALB.

In addition, we further analyzed the correlations between key metabolites of these metabolite pathways and ALB/MC through Spearman correlation analysis (Figure 5C). We identified that the ALB was positively correlated with glycerophosphorylcholine, choline, glutamate, N-acetylaspartate, glycocholate and glycochenodeoxycholate, while negatively to the uridine, dihydroorotate and 2-deoxyuridine. Meanwhile, MC was positively associated with choline, dihydroorotate, glycochenodeoxycholate, glycocholates, sarcosine and succinate.

## Discussion

This study investigated the metabolomic profiles of COVID-19 patients and found the link between their metabolites and various clinical indicators. Our findings clearly revealed that their general metabolism is still in a state of disorder and exhibited abnormal fatty acid and amino acid metabolism.

A key finding reported by several COVID-19 cohort studies is that more than 100 amino acids and their related metabolites were dysregulated after COVID-19 infection, and the majority of which were significantly downregulated (Shen et al., 2020). Compared to their findings, in our COVID-19 patients, many amino acids and their metabolites were returned to normal, suggested a recovery of dysregulated amino acid metabolism after the cure of COVID-19. However, we still noticed several amino acids and their related metabolites were significantly upregulated, such as alanine, glutamate, aspartic acid, sarcosine, leucine, cysteine, etc. These amino acids are key players in energy metabolism and metabolic homeostasis regulation (Pedroso et al., 2015; Paul et al., 2018; Petersen et al., 2019; Waskiw-Ford et al., 2020). Their upregulation could symbolize post-disease recovery, demonstrating that the body is repairing the damage caused by the infection. In the meanwhile, some amino acids, including kynurenine, arginine and tryptophan, remain suppressed. Interleukin-6 (IL-6) levels were linked to tryptophan metabolism (Thomas et al., 2020). Kynurenine and arginine are essential to the immunosuppressive activity of dendritic cells, which are critical immunomodulators (Mondanelli et al., 2017). Their persistent dysregulation may be the underlying molecular mechanism of long-COVID and requires targeted interventions.

Another frequently described metabolomic characteristic of COVID-19 infection is abnormal lipid and fatty acid metabolism (Shi et al., 2021; Tanner and Alfieri, 2021; Zhang et al., 2022). Our study demonstrated that blood level of short-chain fatty acids returned to normal in cured COVID-19 patients, while the long-chain fatty acids and glycerophospholipids were still disturbed. Long-chain polyunsaturated fatty acids and glycerophospholipids, such as stearidonic acid (Correa et al., 2021) and linolenate (Wang et al., 2020), inhibit pro-inflammatory mediator release and immune cell proliferation, hence regulating inflammatory processes (Zeng et al., 2017; Weill et al., 2020; Correa et al., 2021). COVID-19 infection causes immune response dysregulation, and the continuous abnormalities in LCFA and lipid metabolism showed a long-term immune response problem. The findings are similar to those of COVID-19 survivors 6 months after discharge (Li et al., 2022), indicated that the metabolic disturbance of lipid is associated with the long-term chronic discomfort of COVID-19 healers.

The liver is the primary organ concerning amino acid metabolism, and liver damage or dysfunction has been documented often in COVID-19 patients (Jothimani et al., 2020). The elevated levels of ALT, AST, GGT, and bilirubin are common at COVID-19 onset. As for our cured COVID-19 patients, their serum ALT, AST, GGT, creatinine, TBIL and DBIL returned to normal; ALB was down-regulated. DBIL, TBIL and ALB were negatively associated with glycerophospholipid metabolism, alanine and glycine metabolism, etc. Glycerol phospholipids are the components of bile and bile-responsive chaperone (Lee et al., 2020). Alanine and Glycine may protect against liver injury by attenuating oxidative stress and apoptosis in animal experiments (Maezono et al., 1996; Chen et al., 2013). In addition, ALB was also associated with pyrimidine metabolism that has an intrinsic link with liver lipid accumulation for maintaining normal liver homeostasis (Le et al., 2013). Besides, lymphocytes and monocytes were related to the metabolism of pyrimidine pathways and amino acids such as glycine, serine, alanine, etc. Disorders of pyrimidine pathways may lead to immunological diseases (Vincenzetti et al., 2016). As mentioned earlier, amino acid metabolites participate in immunoregulatory.

In conclusion, our findings from a metabolomic comparison of cured COVID-19 patients and healthy controls demonstrated the presence of residual metabolic anomalies. These offered were helpful for the further exploration of COVID-19 patient management, and provided insights into the molecular mechanism and pathophysiological grounds of long-COVID. Our study, however, has some limitations, including limited sample size and a single sampling point. As a result, more research with bigger sample size and more time points is required.

## Data Availability

The original contributions presented in the study are included in the article/Supplementary Material, further inquiries can be directed to the corresponding authors.
