# Supplementary material for: Using an untargeted metabolomics approach to analyze serum metabolites in COVID-19 patients with nucleic acid turning negative
Source: Front Pharmacol. 2022 Aug 24;13:964037. doi: 10.3389/fphar.2022.964037 (PMC9449332; doi:10.3389/fphar.2022.964037)
Supplement: Supplementary file 1 [file DataSheet1.pdf]

Supplementary Material

1. Comparison of different class metabolites in near discharged COVID-19 and heathy control (Figure S1). Valine, leucine, isoleucine and cysteine were up-regulated, gamma-glutamyl-epsilon-lysine, while gamma-glutamylhistidine and gamma-glutamylglutamine in the near discharged patients with COVID-19 were significantly reduced. Besides, 3-hydroxyhexanoate, 3-hydroxyoleate and 3-hydroxyoctanoate were down-regulated, while heptadecanoate (17:1n7), stearidonate (18:4n3), palmitoleate (16:1n7), and miristate (14:0) were up-regulated. Glucocorticoids, cortisone, and cortisol were significantly down-regulated.

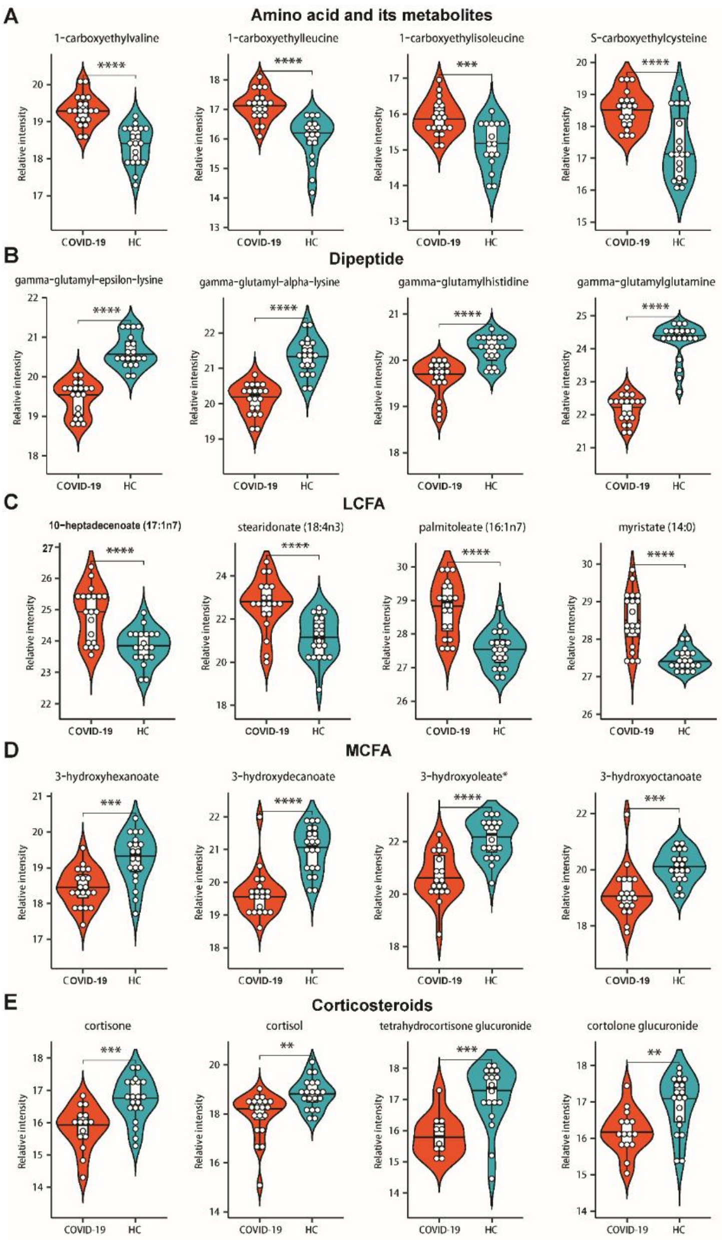

Supplementary Figure 1. Comparison of the relative abundance of different class metabolites in near discharged COVID-19 and heathy control. (A) Relative abundance of Amino acid and its derivatives. (B) Relative abundance of Dipeptide. (C) Relative abundance of MCFA. (D) Relative abundance of Corticosteroids. ⋆ , P value <0.05 ⋆ ⋆ , P value <0.01; ⋆ ⋆ ⋆ , P value <0.001; ⋆ ⋆ ⋆ ⋆ , P value <0.0001.
